# Recent Progress of Carbon Dot Precursors and Photocatalysis Applications

**DOI:** 10.3390/polym11040689

**Published:** 2019-04-16

**Authors:** Kuan-Wu Chu, Sher Ling Lee, Chi-Jung Chang, Lingyun Liu

**Affiliations:** 1 Department of Chemical and Biomolecular Engineering, University of Akron, Akron, OH 44325, USA; kc147@zips.uakron.edu; 2 Department of Chemical Engineering, Feng Chia University, 100 Wenhwa Road, Seatwen, Taichung 40724, Taiwan; sherlinglee0209@gmail.com

**Keywords:** carbon dots, photocatalyst, photocatalysis, top down, bottom up, precursors, polymers, photoluminescence, visible light, electron transfer

## Abstract

Carbon dots (CDs), a class of carbon-based sub-ten-nanometer nanoparticles, have attracted great attention since their discovery fifteen years ago. Because of the outstanding photoluminescence properties, photostability, low toxicity, and low cost, CDs have potential to replace traditional semiconductor quantum dots which have serious drawbacks of toxicity and high cost. This review covers the common top-down and bottom-up methods for the synthesis of CDs, different categories of CD precursors (small molecules, natural polymers, and synthetic polymers), one-pot and multi-step methods to produce CDs/photocatalyst composites, and recent advances of CDs on photocatalysis applications mostly in pollutant degradation and energy areas. A broad range of precursors forming fluorescent CDs are discussed, including small molecule sole or dual precursors, natural polymers such as pure polysaccharides and proteins and crude bio-resources from plants or animals, and various synthetic polymer precursors with positive, negative, neutral and hydrophilic, hydrophobic, or zwitterionic feature. Because of the wide light absorbance, excellent photoluminescence properties and electron transfer ability, CDs have emerged as a new type of photocatalyst. Recent work of CDs as sole photocatalyst or in combination with other materials (e.g., metal, metal sulfide, metal oxide, bismuth-based semiconductor, or other traditional photocatalysts) to form composite catalyst for various photocatalytic applications are reviewed. Possible future directions are proposed at the end of the article on mechanistic studies, production of CDs with better controlled properties, expansion of polymer precursor pool, and systematic studies of CDs for photocatalysis applications.

## 1. Introduction

Carbon dots (CDs) are a new class of carbon-based materials as quasi-spherical and discrete nanoparticles with size typically < 10 nm [1]. Since their first discovery in 2004 [2], a significant amount of research has been conducted on this unique type of carbon-based nanoparticles due to their luminescence properties, photostability, nanoscale size, good solubility in water, low toxicity, biocompatibility, chemical inertness, and low cost. Carbon dots are considered potential candidates to replace traditional fluorescent semiconductor quantum dots, which have serious limitations, such as toxicity related to heavy metals, potential health and environmental hazard, and high cost. Carbon dots have been used for a wide range of applications in biomedical areas such as bioimaging [3], drug delivery [4], gene delivery [3,5], and biosensing [6], and non-biomedical areas such as photocatalysis [7] and optical devices [8].

Photoluminescence (PL) is one of the most appealing characteristics of carbon dots. Region of PL emission spectra of carbon dots is wide and can range from ultraviolet to visible to near infrared. It is generally believed that the surface defects on CDs, behaving as excitation energy traps, or quantum size effect of CDs are responsible for their PL behaviors, although the exact mechanism is still unclear. Either electron donor or acceptor in carbon dots can fulfill photoluminescence. Photoinduced CDs are both excellent electron donors and acceptors, thus can be quenched efficiently by either electron acceptors or donors [9]. Besides the down-converted PL properties, some CDs exhibit distinct up-converted characteristic. The up-converted emission of CDs is believed to be attributed to the two- or multi-photon active process, in which the simultaneous absorption of multiple photons leads to the emission of light at a shorter wavelength than that of the excitation [10,11,12]. Another theory proposes that the upconversion PL originates from the anti-Stokes transition with constant energy difference between π and σ orbitals, as evidenced by the constant energy shift between the emission and excitation light [13]. The up conversion property of CDs has been utilized for unique applications such as deep-tissue imaging [14] and visible-light-driven photocatalysis [7,15]. For example, the composite CD/TiO_2_ photocatalysts have been developed to harness the visible spectrum of sunlight, showing significantly enhanced photocatalytic activity than the unmodified TiO_2_ [7,15,16].

While the existing reviews summarized the synthesis, properties, mechanism, and broad applications (e.g., imaging, sensing, drug delivery, optics, and catalysis) of CDs [9,17,18,19,20,21,22], this review emphasizes the broadness of precursors forming fluorescent CDs, especially the polymeric materials (both natural and synthetic), and recent advances of CDs for photocatalysis applications. Among 50 references on photocatalysis applications of CDs cited in this review, 86% were published during the past five years.

**Figure 1:**
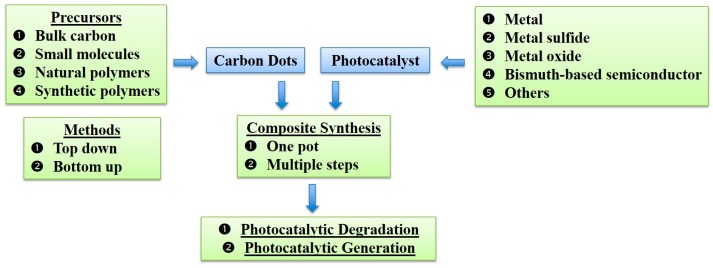
Overall structure of the article.

The article is structured as shown in Figure 1. First, we briefly summarize the major CD fabrication methods in Section 2. An intensive review of small and polymeric precursors for CD formation is then presented in Section 3. Synthesis of the CDs/photocatalyst composites is subsequently discussed briefly in Section 4. In Section 5, we thoroughly review the recent work on applying CDs in the photocatalysis filed. Conclusions and future perspectives are finally discussed.

Carbon dots have been described with different terms in current literature, including “Carbon nanodots”, “C-dots”, “carbon nanoparticles”, or “carbon quantum dots”. In this review, the term “carbon dots (CDs)” will be consistently used.

## 2. Synthesis Methods of Carbon Dots

**Figure 2:**
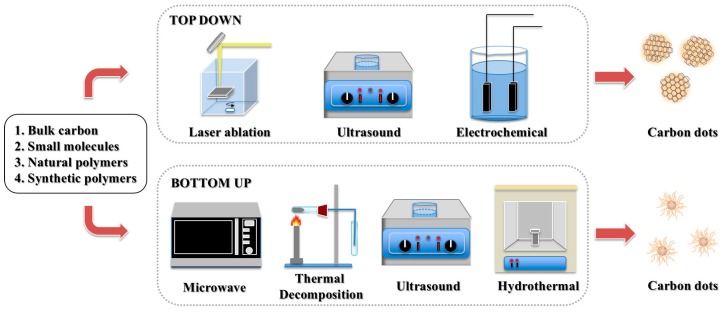
Common top-down and bottom-up approaches to synthesize carbon dots.

Carbon dots can be generated using top-down or bottom-up methods (Figure 2). For the top-down approach, CDs are formed by breaking down larger carbon structures such as graphite [16,23,24], graphene [15], or even fullerene [25]. Conversely, the bottom-up approach generates CDs by decomposition/fusion/carbonization of small or large precursors.

The first CDs, accidentally discovered in 2004, were actually generated with a top-down arc-discharge method using graphite electrodes, when Xu et al. purified the single-walled carbon nanotubes using electrophoresis [2]. More commonly used top-down methods in recent years include laser ablation [10,26,27,28], ultrasonic treatment [15] and electrochemical oxidation [16,23,24,29,30]. The size and nanostructure of CDs are controllable by the one-step route. However, top-down methods often need expensive and complicated equipment, hash reaction conditions, or tedious process, while the quantum yield (QY) and biocompatibility of the resulting CDs are not satisfying. Therefore, these approaches are often followed by chemical treatment to passivate or modify CD surfaces.

The most commonly used bottom-up approaches to produce CDs include microwave approach [31,32,33,34], thermal decomposition [35,36], ultrasonic treatment [37,38], and hydrothermal treatment [39,40,41,42], among others (e.g., pyrolysis [43]). This route utilizes various small or large precursor materials, including both natural and synthetic polymers. Compared to the top-down route, the bottom-up process is facile, cost effective, and environmentally friendly (i.e., no need of harsh reagents or conditions), with a large selection of available precursors. Optical properties of CDs can be easily tuned by changing reaction conditions. Notice that the ultrasonic treatment can be either top-down or bottom-up, depending on the precursor type.

### 2.1. Top-Down Methods

#### 2.1.1. Laser Ablation

Laser ablation of a macro carbon target in liquid or vapor is a simple way to synthesize carbon dots. High laser energy is transferred to the target to break bonds and ablate CDs off the surface. Compared to the chemical methods which are usually time-consuming and need careful control, the laser ablation is a quick and efficient process to produce CDs.

Reyes et al. [28] synthesized CDs through laser ablation of a solid carbon target in acetone. Two ablation parameters, laser wavelength (355, 532, 1064 nm) and ablation time (150, 300, 600, 900 s), were varied and correlated with the morphological and optical properties of the produced particles. It was found that the 355-nm ablation wavelength led to CDs with size less than 5 nm, whereas large agglomeration formed under longer laser wavelengths. The PL emission was compromised when increasing the ablation time, evidenced by decreased PL intensity and broader red-shifted peak. PL intensity also decreased at longer laser wavelength. The highest emission quality was observed using 355-nm ablation for 150 s. Consistently, another work showed that laser ablation, at wavelength of 1064 nm, of graphite target in ethanol generated carbon particles with large size of 200–500 nm and flower-like cluster structure [27].

Carbon dots were also produced by laser ablation (1064 nm) of a graphite target in the presence of water vapor with argon as carrier gas at 900 °C and 75 kPa. The as-produced samples showed no detectable photoluminescence [10,26]. The CDs were then treated with acid, passivated with polymers (polyethylene glycol or poly(propionylethyleneimine-co-ethyleneimine)), and centrifuged to remove large aggregates. The resultant CDs were around 5 nm in diameter and emitted bright luminescence [10,26].

#### 2.1.2. Ultrasonic Treatment

With the alternating low-pressure and high-pressure waves in liquid, the ultrasound treatment generates high-speed liquid jet and strong hydrodynamic shear force, which can cut and deagglomerate bulk carbon materials (e.g., graphene sheets) into nano-sized CDs. The synthesis mostly happens under room temperature instead of high temperature. For example, Zhuo et al. reported an ultrasonic method to generate CDs with diameters of 3–5 nm from graphene [15]. Interestingly, the as-prepared CDs showed both excitation-independent downconversion and upconversion PL behavior, with emissions located at *ca.* 407 nm when excitation wavelength ranged from 240 to 340 nm and from 500 to 700 nm. When combining these CDs with TiO_2_ (a widely used photocatalyst with limited activity under visible light), the photocatalysis efficiency to degrade methylene blue under visible light was much more improved than pure TiO_2_.

#### 2.1.3. Electrochemical Oxidation

Electrochemical synthesis of CDs uses bulk carbon sources as the starting materials. With a stable external voltage applied between two electrodes, the process involves only a simple one-step reaction.

An alkali-assisted electrochemical process was developed to synthesize CDs with size of 1.2–3.8 nm, using graphite rods as both anode and cathode and NaOH/ethanol as electrolyte, with current intensity of 10–200 mA/cm^2^ [16]. In contrast, the acidic electrolyte (H_2_SO_4_/ethanol) led to no CD formation. Similarly, Liu et al. generated CDs from electrochemical oxidation of the graphite working electrode, using mixed ethanol/NaOH/H_2_O as the electrolyte [24]. Application of a potential of 5 V to the working electrode for 3 h led to CDs with average diameter of 4 nm and quantum yield of 11.2%. Interestingly, the CDs displayed excitation-dependent PL intensity but excitation-independent emission wavelength. The CDs were used to specifically detect Fe^3+^ and image cells.

The Huang, Liu, and Kang groups generated CDs by applying static potential (15–60 V) to two parallel graphite rods inserted in ultrapure water, followed by filtration and centrifuge [23,29,30]. Notice that the electrochemical process took 5 to 10 days. CDs were then used to form composites with various metal (Ag, Au, and Cu) nanoparticles for selective oxidation of cyclohexane under visible light [23,29], or hybrid with cadmium sulfide (CdS) nanosheets for photocatalytic water splitting [30]. Notice that many of the CDs applied in the photocatalytic research is based on the electrochemical method from graphite.

### 2.2. Bottom-Up Methods

#### 2.2.1. Microwave Approach

The microwave method utilizes microwave energy to break chemical bonds and carbonize precursors, by exposing them to electromagnetic radiation in the microwave frequency range. This simple method provides outstanding characteristics such as short reaction time, mild reaction condition, cost effectiveness, and low energy consumption. However, it is difficult to finely control the synthesis process (e.g., irradiation power and absorption properties of precursors) with this technique, which could lead to poor reproducibility and low quality of CDs. CDs may have irregular shape, large size distribution, and aggregation since the precursor solution is completely dried eventually.

Zhang and colleagues reported a heterogeneous synthesis process to obtain N-doped CDs, by reacting mixture of calcium citrate and urea under 800-W microwave for 5 min [34]. The resultant CDs emitted yellowish-green fluorescence both in solid and aqueous states. So et al. [31] produced blue luminescent CA-18 CDs (from citric acid and ethylenediamine, with total pyrolysis time of 18 min) and CJ-14 CDs (from citrus calamansi juice and ethylenediamine, with 14-min pyrolysis), using 630-W microwave at 2 min repetitive heating. The latter had much lower production yield and was less uniform and more toxic to bean growth. Huo et al. [32] synthesized CDs by microwaving an aqueous solution of citric acid and ethylenediamine for 10 min at 800 W. The obtained CDs (light yellow) was further used to modify cadmium selenide (CdSe) photocatalyst to degrade tetracycline hydrochloride (an antibiotic pollutant), with the composite photocatalyst showing enhanced degradation efficiency. Liu et al. [33] developed a one-step microwave-assisted method to synthesize green luminescent CDs, by using sucrose as the carbon source and diethylene glycol as the high boiling point reaction medium (in replace of water). The CDs were obtained within one-minute microwave and exhibited excitation-independent PL emission and upconversion PL property.

#### 2.2.2. Thermal Decomposition

Wang et al. [35] produced small CDs (average size of 0.7–1 nm) with high QY (~11%) and excitation-independent blue fluorescence, by thermal decomposition (i.e., thermolysis) of citric acid, with temperature of 180~200 °C and reaction time of 20~30 min. Longer reaction time (>40 min) or higher reaction temperature (230 and 270 °C) favored the formation of larger inhomogeneous particles, which showed excitation-dependent low QY emission. The proposed reaction mechanism is that the citric acid molecules first condense into sp^2^ domains forming nucleus, then crosslink and stack to form larger particles as the reaction proceeds. The small CDs were used as a sensing material for Fe^3+^ with good selectivity and high sensitivity down to 0.3 ppm.

In another work, an ionic liquid-assisted thermal decomposition approach was used to synthesize CDs, using L-cysteine as the precursor and ionic liquid of 1-butyl 3-methyl imidazolium bromide ([bmim]Br) as solvent [36]. L-cysteine dissolved in [bmim]Br was heated at 240 °C for 5, 10, 20, or 60 min, filtered through a porous membrane, and further purified by silica gel to obtain CDs with average size of 34, 48, 84, and 137 nm, respectively. Such large particle size compared to other work is likely due to the high polymerization efficiency under the homogeneous phase provided by [bmim]Br. It was also reported that as the reaction time increased, the PL spectra showed gradual wavelength red shift. The as-prepared crude product was explored for solar cells and other optoelectronic applications.

#### 2.2.3. Ultrasonic Treatment

Bare polyethylene glycol (PEG, Mn = 300) was treated with simple ultrasound irradiation at room temperature for 1, 2, 3, or 4 h without additional solvents, achieving CDs with size of 2-6 nm and quantum yield of up to 14% [37]. The PL emission intensity gradually increased with the increasing synthesis time.

A one-step alkali or acid assisted ultrasonic method was reported to synthesize CDs by ultrasonically treating a glucose aqueous solution for 4 h [38]. The energy of ultrasonic waves caused glucose polymerization, carbonization, and formation of CDs with size less than 5 nm and quantum yield ~7%. Not only the CD emission covered the entire visible to near infrared (NIR) range, but also the obtained CDs had two unique features: NIR emission under NIR excitation and up-conversion luminescence.

#### 2.2.4. Hydrothermal Approach

The hydrothermal synthesis is a process to convert organic compounds in water to structured carbons under high temperature and high pressure. It is one of the simplest and most attractive methods for the preparation of CDs, owing to low cost, cheap apparatus, simple one-step preparation, low energy consumption, and eco-friendly reaction process. In a typical process, the precursor solution is sealed into a Teflon-lined autoclave and heated at 100 to 220 °C for hours. Large particles are then removed to obtain nano-sized carbon dots.

Tao et al. synthesized polymer CDs from polyacrylic acid and ethylenediamine via a one-pot hydrothermal treatment, which emitted both fluorescence and phosphorescence at room temperature [39,40]. Grass was hydrothermally treated for 3 h to produce fluorescent CDs [41]. Increased reaction temperature led to a decrease in particle size and an increase in QY. At temperature of 150, 180, and 200 °C, the diameter range of obtained CDs was 18–22, 3–5, and 2–3 nm, respectively, and the QY was 2.5, 5.1, and 6.2%, respectively. The CDs were used to selectively detect Cu^2+^ ions in buffer (with a detection limit of 1 nM) and in real lake water. A similar study was done by the same research group to make CDs (2–4 nm) from pomelo peel with QY of 6.9%, which were used for sensitive detection of Hg^2+^ ions [42].

In fact, the hydrothermal approach has been the most common approach to produce CDs from a broad range of natural and synthetic polymers. A detailed discussion will follow later in the Section 3.2 and Section 3.3.

### 2.3. Factors Influencing PL Properties of CDs

As seen in some work discussed above, the reaction conditions when synthesizing CDs (e.g., reaction time, temperature, electrochemical potential, and solvent type) drastically impact the intrinsic properties of CDs, which in turn correlate with the PL behaviors of CDs. More examples are provided here.

For instance, PL intensity of CDs, derived from peach gum polysaccharide, enhanced when the hydrothermal reaction time increased from 0 to 12 h [44]. CDs made from peanut shells exhibited higher quantum yield when the pyrolysis time increased from 1 to 4 h [43]. The PEG-derived CDs showed gradually increased PL emission intensity with the increasing ultrasound irradiation time from 1 to 4 h [37]. CDs derived from citrus pectin showed stronger fluorescence intensity when the hydrothermal temperature (100, 120, 150, and 180 °C) increased [45]. In general, these studies show that the PL intensity or QY of CDs can be up tuned by adjusting the treatment time or processing temperature of the precursors.

Different potentials applied to a graphite working electrode (3, 5, or 7 V) resulted in CDs with average size of 2.9, 4.0, and 5.2 nm and QY of 9.5%, 11.2%, and 4.6%, respectively [24]. Solvothermal treatment of citric acid and urea in dimethylformamide leads to CDs with carbonyl and hydroxyl surface groups with excitation-dependent PL, whereas the hydrothermal reaction from the same precursors led to CDs with amine surface groups with excitation-independent down- and up- conversion PL behaviors [46].

Increased CD size typically leads to red shift of the emission peak or weaker luminescence. Li et al. [16] reported size-dependent emission, with small CDs (centered at 1.2 nm) giving UV light emission, medium CDs (1.5–3 nm) emitting visible light, and large CDs (centered at 3.8 nm) emitting near-infrared light. Larger carbon particles (30–50 nm) with the same passivation were found to be much less luminescent than smaller CDs of ~5 nm [26]. Similarly, chitosan-derived carbon nanoparticles with diameters of 30-40 nm exhibited much weaker fluorescence than the 5-nm CDs [47].

Isnaeni et al. synthesized CDs from microwave-assisted treatment of commercial white sugar and observed that as the CD concentration increased, PL intensity increased and peak wavelength shifted to longer wavelength, which was presumably attributed to energy transition at the CD surfaces [48].

Surface states of CDs significantly affect their luminescence properties. An increase in the degree of surface oxidation of CDs with similar particle size caused red shift of their PL emission [49]. Xu et al. [46] developed CDs with engineered surface states (such as C=O, C–OH, or C–NH_2_) and different energy levels, which significantly influenced their optical absorption and PL behaviors. Furthermore, improved surface passivation with more surface coverage by the polymeric passivation agents led to more emissive CDs with higher quantum yield [26].

Depending on the precursors and experimental conditions, CDs could have different morphological structures (e.g., amorphous or crystalline core). The CDs obtained by an alkali-assisted electrochemical method, for example, had clear crystal structures and exhibited both up- and down-converted PL and photo-induced electron transfer ability [50].

## 3. Materials for Carbon Dot Synthesis

Besides the carbon-based bulk materials (e.g., graphite), carbon dots have been generated from both small molecule precursors and macromolecules (natural or synthetic).

### 3.1. Small Precursors

During the early stages of carbon dot studies, much efforts were made to derive CDs from small molecule precursors. A pair of precursors were frequently employed, one serving as carbon source, the other as nitrogen source or passivation agent. Typical carbon sources include citric acid, citrate salts, and acrylic acid, whereas nitrogen sources are more diverse (Table 1).

Hydrothermal treatment of citric acid and ethylenediamine led to CDs with QY as high as 80%, under optimized reaction conditions (e.g., amount of reactants, temperature) [51], whereas if the nitrogen source (e.g., ethylamine, n-heptylamine) bares only one amine, the QY was below 10%. The CDs were applied as printing inks, CD/polymer nanocomposites, or a reagent to detect Fe^3+^.

Three types of CDs (named e-CDs, h-CDs, and t-CDs) were synthesized by hydrothermal reactions of citric acid and three different nitrogen precursors (ethylenediamine, hexamethylenetetramine, and triethanolamine), resulting in the photoluminescence QY of 53%, 17%, and 7% respectively [52]. It was proposed that molecular fluorophores, likely attached to the CDs, led to the stronger emission of e- and h-CDs, besides the contribution of surface state and size.

In Wang‘s work, three kinds of CDs were synthesized from hydrothermal reaction of citric acid and three linear amine analogues (ethylenediamine, diethylenetriamine, and triethylenetetraamine) in different molar ratios [53]. Under the optimized ratio, the highest PL QY achieved for each CD was 69.3%, 68%, and 33.4% respectively. The first two CDs suffered more severe photobleaching under prolonged UV excitation, whereas the last one exhibited excellent PL stability, but at the expense of QY. The CDs were utilized to image Hela cells and subcutaneous injection in mice.

Li et al. [4] developed green-emitting CDs (2–6 nm, QY of 36%) from citric acid and urea by microwave synthesis, as a targeted and trackable drug delivery agent for localized cancer treatment in a liver cancer mouse model. The CD-DOX conjugates, formed by electrostatic attraction between CDs (carboxyl-rich) and cancer drug Doxorubicin (DOX, amine-containing), showed selective killing effect to cancer cells but were safe to normal cells, attributed to the pH difference between cancer and normal cells and pH-triggered DOX release.

Zhang et al. synthesized the fluorescent CDs from acrylic acid and ethylenediamine by microwave-assisted pyrolysis [54]. Subsequent surface vinylation led to the polymerizable CDs (QY = 30.5%), which were copolymerized with several model monomers to form fluorescent polymers in solution, hydrogel, or solid state.

Hydrothermal treatment of p-phenylenediamine and urea led to a mixture of CDs with different colors of fluorescence, which were then critically separated by silica column chromatography [49]. The purified eight CD fractions emitted excitation-independent fluorescence with a gradient of colors from blue to red under a single-wavelength UV light. With similar particle size (~ 2.6 nm), the PL red shift was thus ascribed to the increasing degree of surface oxidation of CDs, leading to reduced band gap, rather than the quantum size effect. The CDs in different colors were used for in vitro cell imaging, whereas the red-emitting CDs were capable of in vivo imaging with strong penetrating fluorescence.

Not only from dual precursors, CDs have also been prepared from single small molecules (Table 2), such as amino acids [6,36,55], small carbohydrates [38,48,55], or other low molecular weight chemicals [7,8,35,51,55]. For example, histidine was used to prepare CDs (3–5 nm, QY of 10.7%) by hydrothermal approach [6]. A fluorescence resonance energy transfer (FRET) system was constructed between these amino-carrying CDs and citrate-stabilized gold nanoparticles (AuNPs) for optical detection of melamine, with the detection limit of 36 nM. Addition of melamine to AuNPs prior to CDs prevented the fluorescence quenching effect of AuNPs to CDs, resulting in the gradually increased fluorescence. In another work, isomers of phenylenediamines (*p-*, *o-*, and *m-* types) were solvothermally treated to achieve CDs (d ~ 10, 8.2, and 6.0 nm, respectively) emitting red, green and blue lights (RGB, the three primary colors) at the same 365-nm excitation [8]. Different PL characteristics of three CDs were hypothetically attributed to their particle size and nitrogen content differences. The CDs showed multicolor cell imaging capability and have potential for deep-tissue imaging due to their two-photon up conversion PL feature. Flexible full-color emissive films were realized by doping polyvinyl alcohol films with mixture of these CDs in appropriate ratios.

Among various reported low molecular weight precursors, the combination of citric acid and ethylenediamine appears to result in CDs with highest photoluminescence quantum yield up to now [51]. In general, CDs prepared from sole small precursors show weaker emission compared to those from dual small precursors.

### 3.2. Natural Polymers and Biomass

CDs have been developed from many natural polymers such as polysaccharides and proteins (Table 3). Chen et al. [56] synthesized CDs, with blue emission under UV excitation, by hydrothermal treatment of lignin at 180 °C, in the presence of H_2_O_2_ which produced hydroxyl radicals (.OH) as the oxidizing agent to break the carbon-carbon linkages of lignin. The treatment time varied from 10 to 60 min, with the maximum CD luminescence occurring at 40 min. The resultant CDs (2–10 nm) exhibited low cytotoxicity, high water solubility, superior photostability than fluorescein isocyanate and CdTe quantum dots, and excellent permeation into Hela cells. By hydrothermal treatment of chitosan at 180 °C for 12 h, CDs (4–7 nm) with remarkably high QY of 43%, excellent photostability, and low cytotoxicity were synthesized and used as a cell imaging agent [47]. The high QY might be related to the inherent nitrogen-doping chitosan precursor. CDs of ~ 7 nm, fabricated from the xylan and NH_4_OH mixture via hydrothermal carbonization at 200 °C for 12 h, showed QY up to 16%, both down- and up-conversion PL, and were applied to image embryonic stem cells [11]. The dilute NH_4_OH solution served as both solvent and passivation agent. Pectin from citrus peel, a natural water-soluble polysaccharide, was hydrothermally treated in the presence of NaOH at different temperatures (100, 120, 150, and 180 °C) for 2 h to form CDs [45]. Fluorescent CDs were obtained even at low temperature of 100 °C, with stronger fluorescence intensity at higher temperatures and no visually carbonized precipitates even at 180 °C. Without NaOH, no fluorescent CDs were formed at 100 and 120 °C and significant black precipitates occurred at 150 °C and above. The CDs derived from the NaOH-mediated pectin hydrogel at 180 °C had average size of 2.7 nm, QY of ~1.1%, good photostability, and no cell toxicity even at 1 mg/mL, and were used to image HEp-2 cells.

Two examples are given here to illustrate the pure protein-derived CDs. Silk fibroin-based CDs (~ 6 nm), synthesized via microwave irradiation (300 W) at 200 °C for 20 min, showed strong blue emission, water dispersity, quantum yield of ~15%, low cytotoxicity, good cell penetration for imaging, and clearance from the animal body within 18 h with no long-term toxicity [57]. CDs with average size of 1.7 nm were obtained by simple hydrothermal treatment of aqueous gelatin solution at 200 °C for 3 h, with synthesis yield of 38.6% and high QY of 31.6% [12]. These CDs, with excitation-dependent down-converted and distinct up-converted PL properties, photostability (no bleaching after 8-h continuous UV irradiation), free dispersity in water and polar solvents, and low cytotoxicity, were explored for cell imaging and optic applications. In the same work, luminescent CDs were also obtained by treating proteases (papain, trypsin) in ethanol at 180 °C.

Not only the pure polysaccharides or proteins were used to make CDs, the use of crude natural materials (including biomass) to make CDs has recently attracted much attention due to the renewable resources and no chemical involvement. For example, hydrothermal treatment of peach gum polysaccharide at 180 °C for 12 h led to the formation of amphibious CDs (2-5 nm), which had excellent solubility and strong photoluminescence both in water and in common organic solvents [44]. These CDs were blended with other water soluble or non-soluble polymers to fabricate nanocomposite films that were light-emitting and highly transparent. CDs with average size of 9 nm were produced by microwaving (800 W) the cashew gum solution for 30-40 min, achieving synthesis yield of 17% and quantum yield of 8.7% [58]. Peanut shells, which are rich in fiber, were used to make fluorescent CDs (1.8–4.2 nm) with QY of 10.6% and higher photostability than rhodamine B [43]. The CDs were synthesized via a simple one-pot pyrolysis treatment at 400 °C for 4 h and successfully applied for Cu^2+^ detection, with a limit of detection of 4.8 μM, ascribed to the ions’ fluorescence quenching effect. Fluorescent CDs from sweet potato extract, realized by hydrothermal treatment at 180 °C for 18 h, had QY of 8.64 % and average size of 3.4 nm [59]. The CDs were successfully applied to image Hela and HepG2 cells and selectively detect Fe^3+^ with a linear range from 1 to 100 μM and detection limit of 0.32 μM.

Cow milk, a carbon source consisting mainly of proteins, sugar, and lipids, was hydrothermally treated at 180 °C for 12 h to form CDs (1-5 nm, yield ~ 34.5%), which were further extracted by ethyl acetate to obtain amphiphilic CDs (ACDs, yield: 18%) [60]. The ACD-Ag nanocomposites were then formed by reacting ACDs (as both reducing agent and support) with AgNO_3_ under UV, which were used as an antibacterial agent alone or incorporated into poly(methyl methacrylate) film to kill *S. Aureus* and *E. Coli*. The ACD-Ag nanocomposites showed better biocidal effect compared with AgNO_3_ at the same silver concentration. Egg white was hydrothermally treated at 220 °C for 48 h, leading to fluorescent CDs with average size of 2.1 nm, high QY of 61%, and synthesis yield of 31% [61]. PL of CDs did not change even after 12-h UV irradiation. The CDs were used to image Hela cells, selectively detect Fe^3+^, and prepare thermosensitive light-emitting composites of poly(N-isopropylacrylamide-*co*-vinylimidazole)-CDs microgels. In another work, egg white or egg yolk was directly treated by plasma for 3 min under ambient conditions [55]. CDs from egg white (~3.39 nm) were amorphous with 6% QY, while CDs from yolk (~2.15 nm) had crystalline structure with QY of 8%. The morphological and emission differences were presumably caused by higher lipid content in egg yolk than in egg white. The CDs showed excellent solubility in water and a broad range of organic solvents. Synthesis yield of CDs in this work was only 6% though. Other examples of using natural polymeric precursors (e.g., bagasse, paper ash, plant leaf, and plant peel) to generate CDs were summarized by an earlier paper [58] thus are not detailed here.

As shown in Table 3, hydrothermal treatment is the most widely utilized approach to make CDs from the plant- or animal- derived bio-resources. A general scheme of the process involves hydrolysis of biopolymers to small molecular weight molecules, partial polymerization and carbonization into CD cores, core growth, and coating formation.

### 3.3. Synthetic Polymers

Synthetic polymers, a big pool of human-made polymers with variations in main chain and side chain, have gradually gained attention in recent years for the synthesis of CDs. Compared to the abundance and diversity of small molecule or natural polymer precursors, synthetic polymer precursors studied to date are much less scoped. Nevertheless, different types of synthetic polymers (i.e., positive, negative, neutral and hydrophilic, hydrophobic, and zwitterionic) have been explored to derive CDs for various applications (Table 4).

Notice that the synthetic polymers we discuss here generally refer to the non-conjugated polymers, which show no fluorescence by themselves. CDs made from conjugated polymers, which possess intrinsic fluorescence of the original polymers, are beyond the scope of this review. Also, passivation of pre-formed CDs with polymers is not the focus in this section. We mainly focus on polymers as CD precursors.

Hu group [3] developed CDs (3–4 nm) from hydrothermal treatment of branched polyethyleneimine (bPEI), which was pre-oxidized with ammonium persulfate (APS). The quantum yield of CDs reached up to 54.3%, in contrast to a low QY of 0.7% from bPEI without the APS oxidation treatment, showing potential in bioimaging. The positive zeta potential of the bPEI-based CDs (~23.8 mV) suggests the existence of amino groups and possibility of using the CDs as gene carrier to condense DNA; the CDs were thus used to successfully transfect 293T cells with the green fluorescent protein gene. In another work, polyethyleneimine (PEI, Mw = 1.8 k, 10 k, 25 k) was hydrothermally treated to produce CDs with blue fluorescence [62]. Note that the higher molecular weight led to larger particles (>10 nm). In Kim’s work, CD was synthesized from PEI and glycerol, using a one-pot microwave-assisted method (700 W, 10 min) [5]. The fluorescent CDs and quencher gold nanoparticles (also modified with cationic PEI) were then assembled with anionic pDNA to form the complex for cell uptake. Release of pDNA in cells resulted in fluorescence recovery due to the distance increase between CDs and gold nanoparticles. This strategy provides an effective way to monitor the carrier/pDNA dissociation in a non-labeled manner. Notice in this work, both polymeric and small molecule precursors were simulataneously employed to prepare CDs.

Using polyamindoamine dendrimer as precursor, CDs with QY of 40% were synthesized through a hydrothermal process. They were used for sensitive Fe^3+^ detection, printing ink, or thermosensitive devices when hybrid with poly(N-isopropylacrylamide) [63].

Tao et al. [39] synthesized fluorescent polymer CDs (20–30 nm) with absolute QY of 44% via a one-pot hydrothermal treatment of polyacrylic acid (PAA, M_W_ = 3500) and ethylenediamine (EDA). To understand the fluorescence mechanism, PAA and EDA were individually or co-treated and optical properties of the products were investigated. Results suggest that the fluorescence might be caused by the synergistic effect of PAA’s polymer chains and EDA’s fluorescence centers. More specifically, fluorophores were likely from EDA, while functions of polymer chains were to restrain vibration and rotation of fluorophores and provide a stable chemical environment to enhance and redshift emission. Interestingly, these CDs also emitted phosphorescence at room temperature, associated with the amide or imide structures and the crosslinking-enhanced emission effect by polymer [40]. The production yield of CDs in this work was remarkably high, up to 63.1%. The CDs had average diameter of 5.4 nm and QY of 32.41% in aqueous solution and 28.77% in solid state.

Yang et al. [62] obtained CDs from linear polyvinyl alcohol (PVA) through hydrothermal method, which partially carbonized the dots at center while retaining the wrapping PVA chains. The polymer dots (2–7 nm) had QY of 1.26%, which was not high but adequate for imaging MC3T3 cells. In another study, PVA-derived CDs (3.9 nm), made from hydrothermal approach, was used to fabricate CDs-TiO_2_ hybrid photocatalyst for contaminant degradation [64]. Polyacrylamide was hydrothermally treated for different amount of time, generating CDs (QY of ~12%) with different core sizes (5, 20, 50 nm), for cell imaging application [65]. PEG-derived CDs via ultrasound irradiation, with QY of up to 14%, were incorporated into a TiO_2_ photocatalytic system for improved detoxification of organic dyes [37].

Synthesis of luminescent CDs from hydrophobic synthetic polymers has been rare. In Aji’s work [66], CDs of polypropylene (PP) from plastic waste bag were produced by heating PP at different temperatures (200, 250, and 300 °C) for 20 min and dispersing them in ethanol, resulting in average particle size of 15, 11, and 8 nm, respectively. It was also observed that the higher the heating temperature, the lower the luminescence intensity. A nontraditional bijective approach was developed by Zhu et al. [67] to generate CDs with one-to-one correspondence between a carbon dot and a polymer nanoparticle precursor. The procedure includes multiple steps: living polymerization of linear poly(methyl acrylate) (PMA), incorporation of enediyne (EDY) moiety into PMA, formation of P(MA-r-EDY) polymer nanoparticles, intramolecular chain collapse within particles by thermally triggered Bergman cyclization, and carbonization at 500 °C. In fact, the final pyrolytic product came from EDY moieties while PMA served as a sacrificial matrix. Therefore, by adjusting the EDY amount in the linear polymer precursor, CDs with different sizes (4.5, 2.1, and 2.0 nm) were achieved. It was found that smaller CDs emitted at longer wavelength, which could not be solely explained by the quantum confinement effect. Subsequent surface modification with PEG significantly decreased the emission intensity (thus QY) although the wavelength peak position still remained same.

Aggregation of nanoparicles is a serious problem, which hinders their applications in drug delivery, imaging, and other fields [68,69]. To address the problem, a unique group of synthetic polymers, named “zwitterionic polymers” which contain equimolar anionic and cationic groups in their structures, were recently explored to derive ultra-stable and fluorescent CDs through a simple one-step microwave heating method (1000 W, 5 min) [70]. The zwitterionic polymers are well known for their superhydrophilic and antifouling properties in resisting nonspecific binding from proteins, cells and microorganisms and have been widely used to protect nanoparticles from aggregation [68,69,71,72,73,74,75,76]. In Wang’s work [70], four zwitterionic linear polymers (Mw = 8–10 k) were explored, including poly(sulfobetaine), poly(carboxybetaine)-2, poly(2-methacryloyloxyethyl phosphorylcholine) (PMPC), and poly(carboxybetaine)-1 (PCB-1). CDs from the first two polymers turned cationic and couldn’t keep their zwitterionic property because of the thermal instability of their repeat units. Notably, PMPC CDs (~12 nm) and PCB-1 CDs (~11 nm) remained zwitterionic and showed excellent colloid stability in various bio-relevant media (phosphate buffered saline, and protein solutions) and under harsh lyophilization conditions. In contrast, CDs made from the traditional citric acid and EDA combination through the same microwave approach aggregated drastically under all conditions and were more cytotoxic than their ultrastable counterparts. QY of the PMPC and PCB-1 CDs was 9% and 7%, respectively. It was hypothesized that the zwitterionic polymer was partially burnt into the carbon core while the remaining part formed a superhydrophilic shell, leading to the ultrastability. Our lab is currently extending the work to other zwitterionic polymers.

## 4. Synthesis of the CDs/Photocatalyst Composite

The CDs/photocatalyst composite can be generated via either one-pot or multi-step synthesis approach.

With the one-pot approach, all starting materials for CDs, photocatalyst, and the composite are mixed together for further treatment. For example, Wang et al. [77] synthesized the hybrid CDs/TiO_2_ photocatalyst on the Ti foil with a one-pot synthesis method, by hydrothermally treating a mixed solution of sodium citrate and hydrogen fluoride containing a Ti foil. CDs (size ~10 nm) were found to uniformly deposit on surfaces of the anatase TiO_2_ crystal particles with {001}, {101}, and {010} facets. The introduction of CDs onto TiO_2_ significantly enhanced the photocatalytic activities to degrade Rhodamine B, under both UV and visible light irradiation. In another study, the nanohybrid catalyst of cerium oxide/carbon dots/reduced graphene oxide (CeOx-CD@RGO) was formed by hydrothermally reacting the mixture of cerium precursor (Ce(NO_3_)_3_.6H_2_O), CD precursor and reducing agent (_L_-cysteine), and graphene oxide [78]. The catalyst was used for dye degradation and water electrolysis under visible light.

More commonly, the CDs/photocatalyst can be synthesized via multi-step process, by incubating CDs and photocatalyst particles (separately prepared earlier) [7,15,16,37], or by treating the photocatalyst precursor solution which contains the pre-made CDs [23,29,30,32,64,79].

For example, the CDs-modified TiO_2_ photocatalyst was prepared by coupling of CDs and TiO_2_ microspheres via a facile sol-gel method [7]. The CDs were pre-made from ascorbic acid via a hydrothermal method, and TiO_2_ microspheres were prepared from the Ti(OBu)_4_ precursor solution.

The AuNP-CD composite was prepared by dropwise addition of HAuCl_4_ solution to the CD solution with vigorous stirring, followed by overnight aging [29]. In Yang’s work [23], the AgNP-CD composite was formed by incubating AgNO_3_ with CDs pre-synthesized by an electrochemical method. The abundant oxygen-containing groups (such as –OH, –COOH, and –C=O) on CD surfaces not only reduced AgNO_3_ to produce Ag nanoparticles, but also prevented particle aggregation. The synergistic catalysis of Ag nanoparticles and CDs and the stabilized Ag nanoparticles led to much improved photocatalytic oxidation of cyclohexane by the composite than the case of Ag nanoparticles alone.

The CDs/CdS nanocomposite was synthesized by first forming a homogeneous solution of sulfur power, cadmium chloride, and electrochemically derived CDs in diethylenetriamine, followed by a hydrothermal treatment [30].

The CDs/ZnO nanocomposite (20–30 nm) was synthesized through a facile hydrothermal method [79]. CDs (1.2–3.8 nm) were first synthesized by an alkali-assisted electrochemical method. The alcoholic solution of Zn(Ac)_2_.2H_2_O and CDs was then hydrothermally treated to achieve the CDs/ZnO nanocomposite, which was further applied to eliminate toxic gas (benzene, methanol) in air under visible light. In Li’s work [64], CDs were first prepared by hydrothermal treatment of PVA precursor. The chemically bonded CDs-TiO_2_ nanohybrid, through Ti–O–C bonds, was then made by in-situ hydrothermal treatment of CDs and Ti(SO_4_)_2_, which showed excellent photocatalytic degradation of methyl orange.

Huo et al. [32] first synthesized CDs from citric acid and ethylenediamine by microwave approach. The CDs/CdSe/rGO composite photocatalyst was then prepared by hydrothermal treatment of a mixed solution of CDs, CdSe precursor, and graphene oxide.

## 5. Photocatalysis Applications of CDs

Photocatalysis is an ecofriendly and sustainable process in which light activates a catalyst to create energetic electrons and holes to actuate further reactions. It has been most commonly applied for the environmental and energy applications. Considering the wide light absorbance, PL properties, and electron transfer ability of CDs, many studies have been carried out to explore their photocatalytic potential. CDs can either act as sole photocatalyst without any co-catalyst or form composite catalysts with other materials (e.g., traditional semiconductor photocatalysts).

### 5.1. Pure CDs as Photocatalyst

Li et al. [50] made CDs (1–4 nm) from graphite rods using an alkali-assisted electrochemical method, which had both down- and up-converted PL. The CDs were effective near infrared (NIR) light-driven photocatalyst for selective oxidation of benzyl alcohol into benzaldehyde, with 92% conversion and 100% selectivity after 12 h of irradiation in the presence of H_2_O_2_. Compared with the TiO_2_ catalyst with similar conversion and selectivity under UV and visible light, the long wavelength NIR light is more moderate and environmentally friendly.

Yang et al. [80] reported that pure CDs without co-catalyst and any modification could act as an excellent photocatalyst to split water to generate hydrogen. CDs were synthesized from multi-wall carbon nanotube oxide via the ultrasonic-hydrothermal process with average diameter of 3.4 nm and relative QY of 2.42%. The hydrogen generation rate of CDs reached 423.7 μmol/(g.h) in pure water under UV irradiation. If methanol (20 vol%) was employed as sacrificial donor, the rate of hydrogen production was improved to 3615.3 μmol/(g.h), 34.8 times higher than that of the commercial Degussa P25 photocatalyst under same conditions.

Owing to their light-harvesting and electron transfer properties, CDs derived from citric acid via thermolysis (~6.8 nm, QY = 2.3%) were used as the sole photosensitizer for solar light-driven hydrogen production, in combination with a molecular Nickel catalyst [81]. Under light irradiation, the photoexcited electrons from CDs were directly transferred to the Nickel catalyst, leading to subsequent reduction of aqueous protons. The hydrogen production reached up to 398 μmol/(g.h).

### 5.2. CD-Containing Composite Photocatalyst

CDs have been combined with other materials to form high-efficiency composite photocatalysts to enhance photocatalytic activity, mostly for environmental (e.g., contaminant degradation) and energy (e.g., hydrogen generation) applications. For example, traditional photocatalysts (e.g., TiO_2_) have wide band gap with response limited to UV light which composes only less than 5% of the solar spectrum, while CDs can harvest visible light and transfer excited electrons into the conduction band of TiO_2_ thus enhancing the photocatalysis efficiency.

#### 5.2.1. Metal/Carbon Dot (CD)

A series of recent studies reveal that the incorporation of metal nanoparticles with carbon dots enhances the catalytic activity due to the synergetic effect. For example, Yang et al. reported the combined silver nanoparticles with carbon dots were able to form an ideal catalyst for the oxidation of cyclohexane [23]. The conversion of cyclohexane achieved up to 58.9%, which was about 5 times higher than pristine Ag nanoparticles, while the selectivity to cyclohexanone reached about 84.6% under visible light irradiation. In addition to silver nanoparticles, gold nanoparticles also attracted a lot of attention by researchers. Sahu et al. recently investigated the performance of gold-doped carbon dots on photoconversion of CO_2_ into small organic acids. The photocatalytic reduction of CO_2_ was enhanced due to the ability of doped gold to harvest and centralize the photoexcited electrons in the carbon dots [82]. Similar trend for the effectiveness of AuNP-CD composites was evidenced in the work by Liu et al. for the oxidation of cyclohexane. It was found that the effectiveness of conversion increased with the reaction time. After 48 hours of reaction, the conversion efficiency reached 63.8%, which was about 13 times higher than the pure AuNPs, and the selectivity towards cyclohexanone was over 99.9% [29].

Although the wide light absorption capability of CDs is employed in photocatalysis application, the electron transfer in CDs is not good enough. Thus, researchers tried to synthesize the transition metal/CDs nanocomposites where the doping of transition metal enhanced the ability to accept and donate electrons. A recent work done by Wu et al. revealed that the photocatalytic efficiency of Cu-N doped carbon dots (Cu-CDs) to oxidize 1,4-dihydro-2,6-dimethylpyridine-3,5-dicarboxylate was 3.5 times higher than pristine CDs [83].

Besides the application in photocatalytic oxidation, carbon dots-transition metal nanocomposites also showed antibacterial properties in a recent investigation on various kinds of transition metal compounds (Cu, Mn, Zn, and Ni compound). It was shown that all the carbon dots-transition metal nanocomposites synthesized inhibited the growth of Gram-negative and Gram-positive bacteria, while the strongest inhibition effect was achieved by the Zn carbon dots [84].

#### 5.2.2. Metal Sulfide/CD

**Figure 3:**
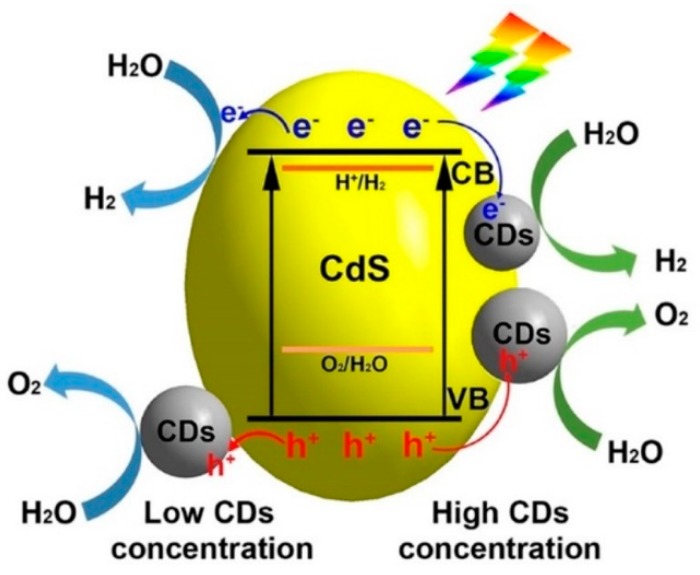
The proposed reaction mechanism of carbon dots-cadmium sulfide (CDs-CdS) under visible light irradiation. Figure adapted from reference [30].

Cadmium sulfide (CdS) is one of the most suitable candidates for photocatalytic applications due to its narrow band gap, excellent chemical and thermal stability and good transport properties. But it suffers from the inherent photocorrosion problem. When incorporating CdS with CDs, the nanocomposites showed significant enhancement on the hydrogen and oxygen production from water splitting as well as the enhanced catalyst stability without sacrificial agents [30]. By controlling the concentration of CDs in the CDs-CdS composite, the hydrogen and oxygen evolution reached about 2.55 and 0.52 μmol h^−1^, respectively. The results did not show an equal stoichiometric ratio of 2:1 (H_2_:O_2_). Furthermore, the CDs-CdS nanocomposite demonstrated higher cyclic stability (8 cycles of catalytic experiments) compared to the previously reported CdS catalyst. Figure 3 shows the proposed reaction mechanism of CDs-CdS, which exhibits good stability after incorporation of carbon dots with CdS, resulting from improved light absorption and separation of the electron-hole pairs. Although this approach still has problems (e.g., low gas production), it creates a new route for developing and studying stable CdS photocatalyst.

Photodegradation of Rhodamine B (RhB) was performed in a study reported by Liu et al., where the CDs can efficiently trap electrons and impede the recombination of photoexcited electrons and holes. After being irradiated for 1 h, the degradation efficiency of RhB by pure CdS reached about 50%, while 1% CDs/CdS nanocomposites enhanced the efficiency to 90%. In short, this investigation proved the significance of CDs-CdS composites in the degradation of dyes [85].

From previous studies on zinc sulfide (ZnS), it is acknowledged that the ability to generate electron-hole pairs upon photoexcitation as well as having highly negative reduction potential of electrons makes it one of the most promising photocatalyst materials. To enhance the photocatalytic activity of CDs, Kaur et al. integrated ZnS with CDs for the degradation of Alizarin red S dye under irradiation of visible light. The as-prepared photocatalyst nanocomposite exhibited significantly high photodegradation activity, 89% within 250 min, which was 1.4 times higher than the pristine ZnS photocatalyst [86].

Molybdenum disulfide (MoS_2_) is widely known to have a graphite-like structure which sandwiches S-Mo-S layers. Owing to this structure, the hydrogen evolution reduction (HER) activity is enhanced because of more catalytically active sites (disulfides S_2_^2−^ and apical S^2−^ sites) for the adsorption of hydrogen existing at the edges of the 2D MoS_2_ crystalline layers. Zhao et al. have demonstrated, with a longer duration of visible light treatment, the HER activity of CDs/MoS_2_ was higher compared to the catalyst with shorter irradiation time. This is due to the transfer of π-electrons from CDs to the S^4+^, in turn providing more S_2_^2−^ and S^2−^ active sites for hydrogen binding, leading to enhanced electrocatalytic activity for HER [87].

Two-dimensional transition metal dichalcogenides (2D TMDs) have gained great attention from researchers, as they possess exceptional characteristics for electrochemical systems and photocatalysis. In a recent study, Atkin et al. reported the synthesis of 2D WS_2_/CD hybrid composites by a simple two-step method of exfoliation followed by microwave irradiation in citric acid solution. The nanocomposite photocatalyst exhibited 30% increase in the rate of photodegradation of Congo Red dye compared to the pristine 2D WS_2_ [88].

#### 5.2.3. Metal Oxide/CD

As seen from previously reported literatures, the fabrication of CDs/ZnO composites and the evaluation of their activity for the photodegradation of dyes have gained substantial attention [89,90]. The significant enhancement of photocatalytic activity is ascribed to the intimate interactions between ZnO and CDs where the CDs absorb light with broader wavelength and then release light with shorter wavelength and excite ZnO to form electron-hole pairs. In a recent study, Ding et al. compared the degradation efficiency of organic dyes by the CDs/ZnO foam composite photocatalyst. In comparison with methyl orange (MO) and Rhodamine B (RhB), CDs/ZnO nanocomposite possessed better photocatalytic activity for the degradation of methylene blue (MB) under visible light irradiation. The decreasing order of degradation efficiency was MB > RhB > MO [91].

Feng et al. also reported the enhanced activity of CDs/ZnO on the photocatalytic degradation of RhB. The degradation efficiency of C-dots modified porous ZnO nanorods was 2.5 times higher than the pristine porous ZnO nanorods [92]. Similar trend is also evidenced for the heterostructured CDs/ZnO [93] and CDs/ZnO nanocomposites [79]. In Yu’s work, the efficiency of CDs/ZnO to degrade gas-phase benzene was 86% after visible light irradiation for 24 h in air at room temperature, much better than the ZnO nanoparticles (~26%) and N-doped TiO_2_ (~60%) [79]. For the treatment of gas-phase methanol, the efficiency was 82, 22, and 55%, respectively, for the CDs/ZnO, ZnO and N-doped TiO_2_. The much improved photodegradation is attributed to the “dyade” structure of the composite and upconverted PL of CDs. Table 5 summarizes the photodegradation applications of various kinds of CDs/ZnO.

For many years, titanium oxide has been one of the most promising photocatalyst as it has good oxidizing ability, environmentally friendly feature, and high thermal and chemical stability. Thus, lots of researchers have studied the TiO_2_ based photocatalysts that bring about high photocatalytic activity. Elkodous et al. optimized the amount of CDs incorporated with macro-mesoporous TiO_2_ (MMPT) for effective wastewater treatment [94]. A CDs@MMPT 30% composite showed the highest photocatalytic activity among CDs@MMPT 29% and CDs@MMPT 32%, because the CDs@MMPT 30% sample had relatively higher surface area. The accelerated formation of pores provided larger number of active sites and helped the diffusion of reactants and the irradiation of light into the inner surfaces of the photocatalyst.

**Figure 4:**
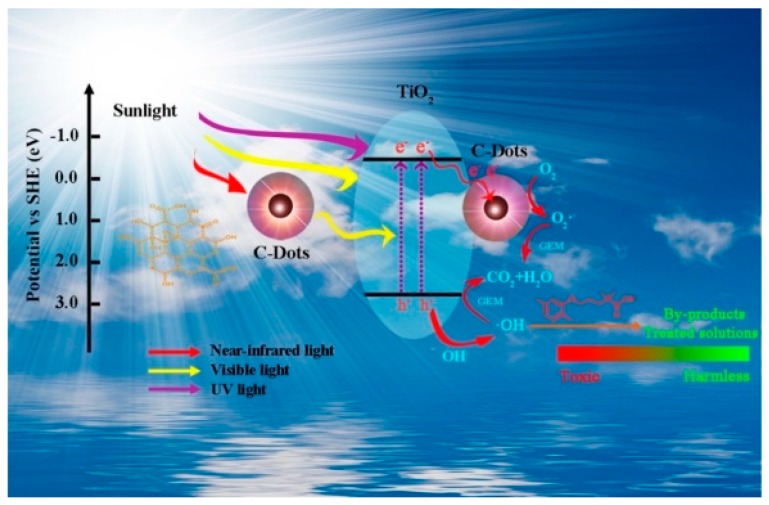
The schematic photocatalytic mechanism of Gembrozil (GEM) degradation by the CDs/TiO_2_ composite under simulated sunlight irradiation. C-Dots: carbon dots. SHE: standard hydrogen electrode. Figure adapted from reference [95].

Besides the application in the wastewater treatment, TiO_2_ photocatalyst is also applied for the treatment of pharmaceuticals and personal care products. From a study conducted by Chen et al., TiO_2_ combined with CDs makes an effective sunlight-driven photocatalyst not only for water purification but also for detoxicating Gembrozil. Even with a very low CD content of 5 wt.%, the reaction rate was 2.3 times faster than the pristine TiO_2_ [95]. Figure 4 depicts the schematic illustration of photocatalytic mechanism of CDs/TiO_2_ under the irradiation of simulated sunlight. CDs were found to have outstanding upconversion photoluminescence. Therefore, after coupling with CDs, lower-energy light (near-infrared or visible light) can be converted to higher-energy light (visible or ultraviolet light) through multiple photon absorption. In addition, since the CDs have good electrical conductivity, the photogenerated electrons can be transferred from the conduction band (CB) of TiO_2_ to CDs to enhance the separation of photogenerated electron-hole pairs. Both the upconversion photoluminescence and the electrical conductivity contribute to improve the photocatalytic activity of the CDs/TiO_2_ composite photocatalyst.

TiO_2_ is also incorporated with N-doped carbon dots to photo-oxidize NOx air pollutants. Martin et al. demonstrated the photooxidation of NO using the composite of TiO_2_ with N-doped carbon dots. The degradation efficiency of NO achieved 27%, which was 2 times higher than pure TiO_2_, and the selectivity for the process was 49.3% under visible light irradiation. The enhancement is due to the increased visible light absorption and hindered recombination of charge carriers [96]. The N-CDs/TiO_2_ composite also showed effective photocatalytic degradation activity when synthesized in the form of hierarchical microspheres, which were built by N-CDs in combination with TiO_2_ nanorods [97]. With this structure, the photodegradation efficiency of RhB dye reached more than 95% within 30 minutes of visible light irradiation, which was 215.7 and 7.3 times higher than the pristine N-CDs and TiO_2_, respectively.

Carbon dots have been incorporated with TiO_2_ into various kinds of structures, such as nanofiber and nanobelt heterostructures. Through a hydrothermal process, CDs were anchored on the nanofibers, which improved the light adsorption and impeded the recombination of electron-hole pairs [98]. Similar structured nanocomposite was also shown in the work by Tian et al., where the CDs/hydrogenated-TiO_2_ (H-TiO_2_) nanobelt heterosturcture harvested light efficiently and promoted charge separation, leading to enhanced photocatalytic activity. The photocatalytic activity of CDs/H-TiO_2_ heterostructures was 3 times higher than that of the pure TiO_2_ nanobelts [99]. In another work, the CDs-TiO_2_ nanohybrids, chemically bonded via Ti-O-C bonds, exhibited a degradation efficiency of 96.7% for MO after UV-Vis irradiation for 8 h. The photocatalytic rate constant of CDs-TiO_2_ was 3.6 and 9.5 times higher than that of TiO_2_ alone and commercial P25, respectively, under visible light irradiation (λ > 420 nm) [64]. In Ke’s work, the degradation efficiency of CDs-TiO_2_ over MB was as high as 90% within 2 h under visible light irradiation, which was 3.6 times higher than that of pure TiO_2_ [7]. Similarly, the photodegradation efficiency of MB after 60-min visible light was up to 97% for CDs/rutile TiO_2_, much high than that for CDs/anatase TiO_2_ (31%), rutile or anatase TiO_2_ (<5%), or pure CDs (~10%) [15]. After visible light irradiation for 25 min, reduction of MB by CDs/TiO_2_ nanocomposite was almost complete, whereas no or little reduction of MB was observed when using pure TiO_2_ (<5%) or pure CDs only (nearly 0%) as photocatalysts [16]. Notice that the CDs in this study showed upconverted PL and were attached to the TiO_2_ surfaces, which allowed efficient visible light harvesting and electron transfer within the composite, thus significantly increasing the photocatalysis activity. In Wang’s work, CDs-TiO_2_ (composite of carbon nanodots and TiO_2_ microcolumns) decomposed almost all the RhB within 75 min of the UV irradiation, while TiO_2_ alone decomposed ~76% of the dye [77]. With the visible light, CDs-TiO_2_ decomposed ~ 77% RhB in 150 min, while pure TiO_2_ showed almost no dye decomposition. The degradation activity of the composite catalyst under visible light almost did not change after 4 reaction cycles. Table 6 summarizes the photodegradation applications of various kinds of CDs/TiO_2_.

#### 5.2.4. Bismuth-Based Semiconductor/CD

**Figure 5:**
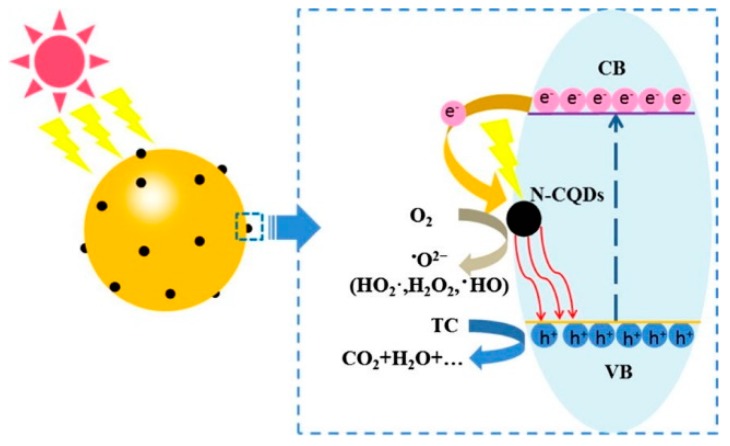
Graphical illustration of possible photocatalytic reaction mechanism of the nitrogen-doped CDs/Bi_2_WO_6_ composite. Figure adapted from reference [101].

The photocatalytic efficiency of carbon dots is frequently enhanced by incorporating CDs with other compounds, ascribed to the broadening of the light absorption region as well as the hindered recombination of electron-hole pairs. Interestingly, when CDs are loaded with Bi_2_WO_6_, the superior photocatalytic activity is attributed to the photo-induced electron transfer and electron reservoir characteristics of CDs. The enhancement portrayed by CDs/Bi_2_WO_6_ composites has been demonstrated in the photodegradation of various kinds of organic pollutants. For example, Wang et al. reported the fabrication of 0D/2D carbon dots on Bi_2_WO_6_ ultrathin nanosheets for the degradation of MO and bisphenol A (BPA) [100]. It was shown that the CDs/monolayered-Bi_2_WO_6_ composite with 3 wt.% CDs had the highest photocatalytic activity for both MO and BPA dyes, with the degradation efficiency about 2-fold and 3-fold higher than that of the pristine m-Bi_2_WO_6_, for MO and BPA respectively. Similar trend of degradation was also exhibited in the N-CDs/Bi_2_WO_6_ [101], CDs/Bi_2_WO_6_ nanocomposites [102] and visible-light-driven CDs/Bi_2_WO_6_ hybrid materials [103]. Figure 5 presents the possible photocatalytic reaction mechanism of N-CDs/Bi_2_WO_6_. The photogenerated electrons can migrate from the CB of Bi_2_WO_6_ to N-CDs, resulting in effective separation of photogenerated electron-hole pairs. Electrons on N-CDs can react with O_2_ to generate •O^2−^ in the solution, leading to enhanced photocatalytic performance.

Recently, Bi_2_MoO_6_ aroused much attention for its captivating properties such as having band gap in the region of 2.5–2.8 eV, chemical stability, low cost, and resistance to corrosion. By incorporating carbon dots with Bi_2_MoO_6_ nanosheets through hydrothermal method, the nanosheet structure of Bi_2_MoO_6_ is expected to enhance the photocatalytic activity. The relationship between structure and photocatalytic activity was investigated by Di et al. [104] They found that the as-prepared CDs modified Bi_2_MoO_6_ showed higher Brunauer–Emmett–Teller (BET) surface area, leading to more photocatalyst-pollutant contact and higher absorption of active species. This is evidenced in the photodegradation of ciprofloxacin (CIP), where a 5-fold enhancement than pure Bi_2_MoO_6_ was shown by 2 wt.% CDs modified Bi_2_MoO_6_. In short, the modification brings several advantages, including more active adsorption sites and photocatalytic reaction centers, wider visible light absorption region, and slower electron-hole recombination rate.

**Figure 6:**
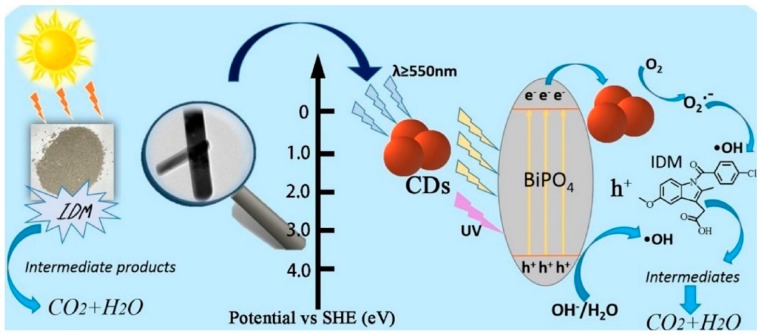
The schematic photocatalytic mechanism of indometacin (IDM) degradation by the CDs-doped BiPO_4_ composite under irradiation of simulated sunlight. Figure adapted from reference [105].

One of the most well-known oxyacid salt photocatalyst, bismuth phosphate (BiPO_4_), has gained lots of research interest owing to its chemically stable structure and excellent electronic and optical properties. It is also reported to display preferable photodegradation of dyes than TiO_2_. However, the application of BiPO_4_ is limited as it exhibits fewer active sites, low quantum efficiency and fast electron-hole pair recombination rate. These limitations can be overcome by loading with carbon dots, which possess the ability to absorb wider range of visible light and excellent up-converted photoluminescence. The enhanced photocatalytic activity was demonstrated by Zhang et al. [105]. The photocatalytic activity of composite photocatalyst with 3 wt.% CD content was 12 times higher than that of pristine BiPO_4_. However, an excess of CD content caused decreased degradation rate. A mechanism was proposed in Figure 6 to interpret the enhanced photocatalytic activity after the introduction of CDs. The photogenerated holes from valence band (VB) of BiPO_4_ oxidize OH^−^ or H_2_O to generate •OH because the VB of CDs/BiPO_4_ is more positive than the standard redox potential of •OH/OH^−^ and •OH/H_2_O. The photogenerated electrons on CDs react with O_2_ to generate O_2_•^−^ in the solution. The formation of •OH, hole, and O_2_•^−^ all contributes to the effective photocatalytic degradation of indometacin (IDM). Similar trend of enhancement was also observed in the work by Di et al. with nitrogen-doped carbon dots/BiPO_4_ materials for the degradation of ciprofloxacin (CIP) [106].

There have been growing appeals for bismuth oxyhalides due to their outstanding photoelectric and catalytic properties. Among numerous BiOX (X = Cl, Br, I) semiconductors, BiOBr has drawn much attention because of its appropriate band gap and photocatalytic activity under visible light irradiation. Nevertheless, the photocatalytic activity is limited because of its less efficient separation of photogenerated electron-hole pairs. To overcome this limitation, Zhao et al. synthesized CDs/BiOBr microspheres via a solvothermal and hydrothermal method [107]. Content of CDs was optimized to achieve the highest activity for the photocatalyic degradation of RhB by the composite photocatalyst, which was 5.3 times higher than that of pristine BiOBr under visible light. Not only the degradation of RhB was enhanced, the photodegradation of p-Nitrotouluene (PNP) was also achieved by the CDs/BiOBr composite, which could not be realized by pure BiOBr. The excellent photocatalytic activity of BiOBr was also reported by Xia et al., where CDs/BiOBr displayed the highest photocatalytic activity for the degradation of two model pollutants, RhB and CIP, when compared with CDs/BiOCl [108].

In addition to BiOBr, BiOI is also studied by researchers due to its potential applications in photocatalysis. BiOI is not only photochemically stable but also has the smallest band gap of about 1.7–1.9 eV among three bismuth oxyhalides (BiOI, BiOBr, BiOCl). Through the fabrication process, the specific surface area of the CDs/BiOI composite was increased as CDs were distributed on the surface of the BiOI microsphere, leading to better adsorption ability than pure BiOI [109]. The photocatalysis enhancement was shown by the composite with 1.5 wt.% of CDs, where the degradation of methyl orange reached 98% in 50 min while the pure BiOI sample only achieved 40%. In short, the addition of CDs, which act as electron-accepting and transport centers, impedes the recombination of charge carries, thus improving the photocatalytic degradation of the dye.

For the past decades, bismuth-based oxides have gained much attention owing to their hybridized valence band by O_2p_ and Bi_6s_. Among numerous bismuth-based oxides, Bi_2_O_2_CO_3_ is a new type of photocatalyst composed of a unique structure with CO_3_^2−^ layers intertwined with [Bi_2_O_2_]^2+^ layers. The photocatalytic degradation efficiency of CDs/Bi_2_O_2_CO_3_ was reported to be as high as 94.45% for methylene blue, which was 1.7 times higher than that of pristine Bi_2_O_2_CO_3_. The degradation efficiency was 61.46% for phenol. The enhancement was proved to be attributed to the upconverted photoluminescence of CDs which enhanced the electron transfer and absorption of visible light [110]. Table 7 presents the CDs/Bi composite photocatalysts with their degradation efficiency, mostly after irradiation of visible light.

#### 5.2.5. Others

Carbon nitride is well-known by its suitable visible light-driven band gap to enhance degradation of pollutants, hydrogen production and CO_2_ reduction. However, the photocatalytic activity is limited by the fast recombination of charge carriers and low visible light absorption. On the other hand, carbon dots are recognized to be able to hinder the recombination of electron-hole pairs and widen the photo-absorption region. Hence, some studies indicate that combining both materials leads to composites with enhanced photocatalytic activity. For instance, Wang et al. reported the N-doped carbon dots/g-C_3_N_4_ composite exhibited improved degradation of indometacin (IDM). With the increasing amount of the NCDs, the composite photocatalytic activity first increased, but then decreased [111]. It is reported that the decomposition of IDM by 1.0 wt.% NCDs/g-C_3_N_4_ composite reached 91.5% while pure g-C_3_N_4_ decomposed only 16% of the pollutant under 90-min visible light irradiation.

Recently, various kinds of fabrication methods were investigated to improve the activity of photocatalysts. For instance, Jian et al. fabricated the nanocomposite of carbon dots/proton functionalized graphitic carbon nitride (HpCN) via an electrostatic self-assembly strategy. The method enabled the formation of intimate 0D/2D heterojunction interface between CDs and HpCN, which improved the electron conductivity of CDs and in turn increased the efficiency for charge separation [112]. The CDs/g-C_3_N_4_ nanocomposite can also be synthesized by using calcination process. Hong et al. fabricated CDs/g-C_3_N_4_ by a low temperature process at 80°C [113], which exhibited the degradation efficiency for RhB and TC-HCl about 3.7 and 1.9 times higher than that of pristine g-C_3_N_4_, respectively. In another study, Zhang et al. fabricated CDs/g-C_3_N_4_ via a simple impregnation-thermal method. It was found that the absorption region for visible light was broadened as the photoluminescence character of CDs was up-converted, leading to the increased production of charge carriers [114]. The enhancement was evident, as the 0.5 wt.% CDs/g-C_3_N_4_ composite demonstrated 3.7 times higher photodegradation rate of phenol than bare g-C_3_N_4_. Similar fabrication method was also reported in the work by Guo et al., where the CDs/carbon nitride hybrid photocatalyst was synthesized under 450 °C [115]. The photocatalytic degradation of MO by the composite photocatalyst reached 90% after 4 h of infrared irradiation, while no degradation of MO occurred when pure carbon nitride was used.

For the removal of NOx, iron-containing catalysts including iron (III) hydroxide (FeOOH) have gained much attention due to their low toxicity, environmental friendliness and favorable band gap. Nonetheless, the photocatalytic performance of FeOOH is limited by its rapid charge carrier recombination rate owing to its poor electrical conductivity. In a recent study done by Huang et al., the CDs/FeOOH photocatalyst demonstrated higher photocatalytic activity than pristine FeOOH [116]. Nearly 22% of the initial nitrogen oxide (NO) was removed by the CDs/FeOOH nanocomposite within 30 min of visible light irradiation, while pure FeOOH showed negligible NO removal. In addition to high photocatalytic activity, the low cytotoxicity and good biocompatability of the nanocomposite ensure minimal environmental effect when applied in large scale.

**Figure 7:**
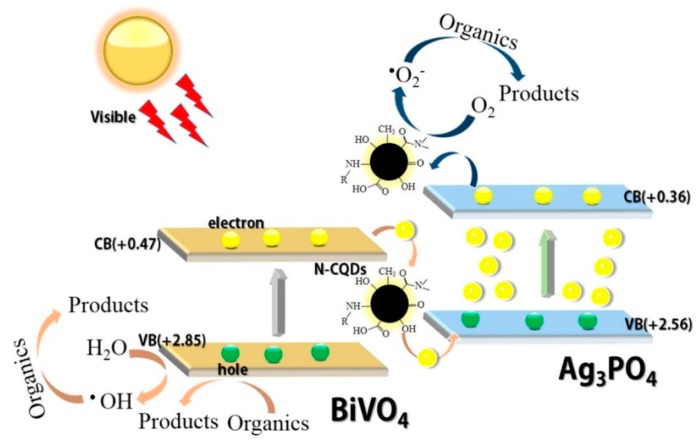
A schematic mechanism of Z-schemed NCDs/Ag_3_PO_4_/BiVO_4_ photocatalyst to degrade organics. Figure adapted from reference [117].

In addition to heteroatom doping, construction of Z-schemed photocatalytic system is also a promising approach to achieve efficient separation of photoexcited charge carriers. Presence of appropriate band gaps between the semiconductors leads to efficient charge separation and more reactive species. Based on a recent study done by Zhang et al., the nitrogen-doped CDs/Ag_3_PO_4_/BiVO_4_ Z-schemed photocatalyst showed high efficiency in the photodegradation of antibiotic, excellent stability and reusability [117]. The degradation efficiency of tetracycline hydrochloride reached as high as 88.9% in just 30 min and remained 79.9% even after five recycling runs. The Z-schemed system enabled greater light-harvesting capacity and enhanced molecular oxygen activation ability. A mechanism proposed in Figure 7 shows the band structure of Z-schemed NCDs/Ag_3_PO_4_/BiVO_4_ photocatalyst and the transfer of photoexcited charge carriers among NCDs, Ag_3_PO_4_, and BiVO_4_. Z-schemed CDs/MoO_3_/g-C_3_N_4_ photocatalyst was also synthesized by Xie et al. for the degradation of tetracycline [118]. With the optimum doping of 0.5% CDs, the Z-schemed CDs/MoO_3_/g-C_3_N_4_ photocatalyst exhibited 3.5 times higher degradation rate of tetracycline than MoO_3_/g-C_3_N_4_. The enhancement is due to the synergistic effects of up-converted PL of CDs, high capacity of charge separation, and the Z-scheme heterojunction structure.

Huo et al. prepared the CDs/CdSe/rGO via a hydrothermal method. With the unique 2D layer structure of rGO, the composite photocatalyst exhibited greatly enhanced photodegradation rate of 90% in 60 min under irradiation of visible light [32]. In short, the incorporation of semiconductors with carbon dots effectively improves the photocatalytic activity, resulting from the synergetic effect between the semiconductors and CDs. Table 8 shows the photodegradation activity of various kinds of CD composites.

## 6. Summary and Perspectives

This article provides a basic review of the common top-down and bottom-up synthesis methods, precursor materials, and photocatalysis applications of CDs. CDs have been successfully derived from numerous precursors including bulk carbons, small organics, natural and synthetic polymers. The photocatalytic activities of CDs, realized either by CDs alone or from hybrids of CDs and other photocatalysts, have been reported in many environmental and energy applications.

Although significant progress has been made in the field of CDs over the past fifteen years since their discovery, it is still facing many challenges. More efforts could be put in future in the following topics:

1. Fundamental understanding of the fluorescence mechanism. Exact mechanism of CDs’ photoluminescence phenomenon is still unclear and requires further investigation.
2. Improve production yield of CDs. With the current methods, production yield of CDs is generally low (<15%) [47,56], since large and less luminescent residues are removed during the purification steps (e.g., centrifuge, filtration).
3. Better control of CD size and homogeneity. Large size distribution leads to broad PL spectrum, complicates the mechanistic studies of CDs, and may impede the condition optimization for the relevant applications.
4. CDs with both high quantum yield and high photostability. Quantum yield of CDs is generally low compared to that of quantum dots except a few cases. The apparently highest QY of CDs up to date, generated from precursors of citric acid and ethylenediamine, originates largely from the molecular fluorophores attached to the CDs instead of CDs themselves, which causes photobleaching [52,53].
5. Increase pool of polymer precursors and elucidate structure-property correlations. Synthetic polymers that have been used to make CDs represent only a very small portion among all. There is a lack of rational design of precursor structures and understanding of their correlations with CD properties.
6. Comparative studies of CDs for photocatalysis. The research of CDs as photocatalysts so far is somewhat qualitative. Systematic work is largely missing to investigate the effect of CD properties (e.g., particle size, concentration, composition, and PL properties) on photocatalysis efficiency and compare different CDs.
7. Photocatalysis applications in real environment. Current literature mostly targets only a few model contaminants (e.g., methylene blue, methylene orange) in pure water. Real-world problems in complex environment have rarely been tackled, for example, degradation of multiple antibiotics and pesticides in river water, lake water, or even soil.
8. CDs with dispersion stability in controlled and complex environment. Carbonaceous aggregation during the synthesis process of CDs is a major setback. CDs also must remain dispersed for practical applications. Particularly for the photocatalysis applications, aggregation of CDs would decrease the surface contact area and increase the recombination rate of electron-hole pairs, causing decreased catalysis efficiency. Surface properties of CDs are thus critical to keep CDs stable (i.e., no aggregation) not only in controlled environment (e.g., water and organic solvents) but also in complex real environment. Stable CDs also potentially increase the reusability of photocatalysts with more cycles.

## Figures and Tables

**Table 1:** Small molecule dual precursors for carbon dot (CD) synthesis. QY: quantum yield.

Carbon Source	Nitrogen Source	QY	Application	Reference
Citric acid	Ethylenediamine	80.6%	Ink, Fe^3+^ detection, CD/polymer composites	[51]
	Ethylamine	8.4%	--	
	n-heptylamine	7.7%	--	
	Urea	19.4%	--	
Sodium citrate	Ethylenediamine	21.6%	--	[51]
Citric acid	Ethylenediamine	53%	--	[52]
	Hexamethylenetetramine	17%	--	
	Triethanolamine	7%	--	
Citric acid	Ethylenediamine	69.3%	Bioimaging	[53]
	Diethylenetriamine	68%	Bioimaging	
	Triethylenetetraamine	33.4%	Bioimaging	
Citric acid	Urea	36%	Drug delivery	[4]
Acrylic acid	Ethylenediamine	30.5%	Fluorescent polymers	[54]
p-phenylenediamine	Urea	up to 35%	Bioimaging	[49]
Calcium citrate	Urea	10.1%	Ink	[34]
Citric acid	Ethylenediamine	1.7%	--	[31]

**Table 2:** Small molecule sole precursors for CD synthesis.

Precursor (Method ^1^)	QY	Application	Reference
Histidine (HT)	10.7%	Melamine sensing	[6]
Cysteine (TD)	--	Solar cells, optoelectronics	[36]
Serine (PT)	Blue fluorescence	--	[55]
Glucose (US)	7%	--	[38]
Glucose (PT)	Blue fluorescence	--	[55]
o-phenylenediamine (ST)	10.4%, green	Multi-color bioimaging Flexible full-color emissive film	[8]
m-phenylenediamine (ST)	4.8%, blue		
p-phenylenediamine (ST)	20.6%, red		
Citric acid (TD)	11%	Fe^3+^ detection	[35]
Citric acid (HT)	7.2%	--	[51]
Ascorbic acid (HT)	--	Photocatalysis	[7]
Ethylenediamine (HT)	3.8%	--	[51]
Acrylamide (PT)	Blue fluorescence	--	[55]
EDTA disodium salt (PT)	Blue fluorescence	--	[55]

^1^ HT: hydrothermal treatment; TD: thermal decomposition; PT: plasma treatment; US: Ultrasonic treatment; ST: solvothermal treatment; EDTA: ethylenediamine-tetraacetic acid.

**Table 3:** Natural polymer-derived CDs, preparation methods, and applications.

Starting Material	Synthesis Method	Application	Reference
Lignin + H_2_O_2_	Hydrothermal, 180 °C, 40 min	Bioimaging	[56]
Chitosan	Hydrothermal, 180 °C, 12 h	Bioimaging	[47]
Xylan + NH_4_OH	Hydrothermal, 200 °C, 12 h	Bioimaging	[11]
Citrus pectin + NaOH	Hydrothermal, 100–180 °C, 2 h	Bioimaging	[45]
Silk fibroin	Microwave (300 W), 20 min	Biomedical	[57]
Gelatin	Hydrothermal, 200 °C, 3 h	Bioimaging, optical devices	[12]
Peach gum polysaccharide	Hydrothermal, 180 °C, 12 h	Optical devices	[44]
Cashew gum	Microwave (800 W), 30–40 min	--	[58]
Peanut shell	Pyrolysis, 400 °C, 4 h	Metal ion detection (Cu^2+^)	[43]
Sweet potato	Hydrothermal, 180 °C, 18 h	Bioimaging, metal ion detection (Fe^3+^)	[59]
Pomelo peel	Hydrothermal, 200 °C, 3 h	Metal ion detection (Hg^2+^)	[42]
Grass	Hydrothermal, 150–200 °C, 3 h	Metal ion detection (Cu^2+^)	[41]
Cow milk	Hydrothermal, 180 °C, 12 h	Antimicrobial	[60]
Egg white	Hydrothermal, 220 °C, 48 h	Metal ion detection, bioimaging, optical devices	[61]
Egg white or egg yolk	Plasma treatment, 3 min	Printing ink	[55]

**Table 4:** Synthetic polymer-derived CDs, preparation methods, and applications.

Polymer	Structure	Synthesis Method	Application	Reference
Branched polyethyleneimine	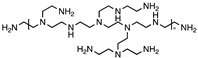	Hydrothermal	Bioimaging, gene delivery	[3]
Polyethyleneimine	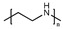	Hydrothermal	--	[62]
Polyethyleneimine (+glycerol)	Same above	Microwave	Gene delivery	[5]
Polyamindoamine dendrimer	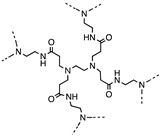	Hydrothermal	Fe^3+^ detection, ink	[63]
Polyacrylic acid (+EDA)	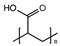	Hydrothermal	Graphic security, information encryption	[39,40]
Polyvinyl alcohol	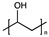	Hydrothermal	Bioimaging	[62]
			Photocatalysis	[64]
Polyacrylamide	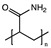	Hydrothermal	Bioimaging	[65]
Polyacrylamide	Same above	Plasma treatment	--	[55]
Polyethylene glycol	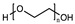	Ultrasonic	Photocatalysis	[37]
Polypropylene	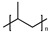	Thermal decomposition	--	[66]
P(methyl acrylate-r-EDY)	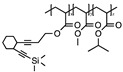	Thermal decomposition	--	[67]
PMPC	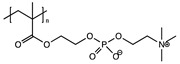	Microwave	Biomedical	[70]
PCB-1	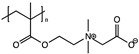	Microwave	Biomedical	[70]

**Table 5:** Photocatalytic degradation activity of CDs/ZnO.

Photocatalyst	Structure	Synthesis Method	Light Source	Model Pollutant ^1^: Degradation Efficiency/Time	Ref (year)
CDs/ZnO foam	nanocomposite	Dispersion in CDs solution	250-W Xe (vis) (λ ≥ 400 nm)	MB > RhB > MO	[91] (2016)
CDs/ZnO	Porous nanorods	Solvent thermal + deposition	300-W Xe (vis) (λ ≥ 420 nm)	Phenol: 94.3%/60 min	[92] (2015)
CDs/ZnO	Heterostructure	Sol-gel + spin coating	18-W UV lamp (vis) (λ = 365 nm)	RhB: 30%/120 min	[93] (2013)
CDs/ZnO	Nanocomposite (20–30 nm)	Hydrothermal	3 of 8-W visible light lamp	Benzene gas: 86%/24 h Methanol gas: 82%/24 h	[79] (2012)

^1^ MB: methylene blue; RhB: Rhodamine B; MO: methyl orange.

**Table 6:** Photocatalytic degradation activity of CDs/TiO2.

Photocatalyst	Structure	Synthesis Method	Light Source	Model Pollutant ^1^	Degradation Efficiency/Time	Ref (Year)
CDs/TiO_2_	Macro-mesoporous nanospheres	Dispersion	300-W halogen lamp (vis)	MB	-	[94] (2018)
CDs/TiO_2_	Composite	Hydrothermal-calcination	350-W arc Xe lamp (vis) (λ < 420 nm)	GEM	89%/8 min	[95] (2017)
N-CDs/TiO_2_	Composite	Hydrothermal	6-W fluorescent lamp (vis) (λ > 400 nm) UV lamp (UV) (λ=365 nm)	NO	27%/120 h (vis) 79.6%/85 h (UV)	[96] (2016)
N-CDs/TiO_2_	Hierarchical microspheres/nanorods	Hydrothermal	500-W Xe (vis) (λ = 420 nm)	RhB	> 95%/30 min	[97] (2013)
CDs/TiO_2_	Nanofibers	Hydrothermal	Natural sunny day (11 a.m. and 3 p.m.)	MB	71%/95 min	[98] (2015)
CDs/Hydrogenated TiO_2_	Nanobelt heterostructure	Hydrothermal + bath reflux	350-W Hg lamp (UV) (λ = 365 nm) 300-W Xe arc lamp (vis)	MO	> 86%/25 min (UV) 50%/25 min (vis)	[99] (2015)
CDs/TiO_2_	Nanohybrid	Hydrothermal	500-W halogen lamp	MO	96.7%/8 h (UV-vis)	[64] (2018)
CDs/TiO_2_	Nanoparticles/microsphere hybrid	Sol-gel method	500-W Xe lamp (vis) (λ > 420 nm)	MB	90%/2 h	[7] (2017)
CDs/rutile TiO_2_	Nanocomposite	Mix + vacuum drying	350-W Xe lamp (vis) (λ > 420 nm)	MB	97%/1 h	[15] (2012)
CDs/TiO_2_	Nanocomposite	Sol-gel method	300-W halogen lamp (vis, λ not specified)	MB	ca. 100%/25 min	[16] (2010)
CDs/TiO_2_	Nanodots/microcolumn composite	One-pot hydrothermal	14 W UV lamp 500-W Xe lamp (vis) (λ > 420 nm)	RhB	ca. 100%/75 min (UV) 77%/150 min (vis)	[77] (2015)

^1^ MB: methylene blue; GEM: Gembrozil; NO: nitrogen oxide; RhB: Rhodamine B; MO: methyl orange.

**Table 7:** Photodegradation efficiency of CDs/Bi composites.

Photocatalyst	Structure	Synthesis Method	Light Source	Model Pollutant ^1^: Degradation Efficiency/Time	Ref (Year)
CDs/Bi_2_WO_6_	0D/2D ultrathin nanosheets	Hydrothermal	300-W Xe (vis)	MO: 94.1%/120 min BPA: 99.5%/60 min	[100] (2018)
N-CDs/Bi_2_WO_6_	Hybrid material	Hydrothermal	300-W Xe (vis) (λ = 420 nm)	TC: 97%/25 min	[101] (2018)
CDs/Bi_2_WO_6_	Nanocomposite	Hydrothermal	500-W Xe (solar light)	RhB: 97%/10 min Phenol: 33.4%/120 min	[102] (2017)
CDs/Bi_2_WO_6_	Hybrid material	Hydrothermal	300-W Xe (vis) (λ = 400 nm)	RhB: ~98%/120 min CIP: 87%/120 min BPA: ~45%/120 min TC-HCl: ~78%/120 min	[103] (2015)
CDs/Bi_2_MoO_6_	Irregular nanosheets	Hydrothermal	300-W Xe (vis) (λ = 400 nm)	CIP: 88%/120 min BPA: 54%/120 min	[104] (2015)
CDs/BiPO_4_	Nanorods	Hydrothermal- calcination	350-W Xe (λ ≥ 290 nm)	IDM: ~ 90%/120 min	[105] (2018)
N-CDs/BiPO_4_	Nanoparticles /nanorods	Ionic liquid assisted solvothermal	250-W high pressure Hg (UV)	CIP: 87.5%/120 min	[106] (2017)
CDs/BiOBr	Microspheres	Solvothermal and hydrothermal	300-W Xe (vis) (λ = 400 nm)	RhB: ~100%/145 min PNP: 26%/320 min	[107] (2016)
CDs/BiOX (X=Br, Cl)	Hybrid nanosheets	Ionic liquid induced	300-W Xe (vis) (λ = 400 nm)	BiOBr: RhB: ~100%/30 min CIP: 44.3%/240 min	[108] (2016)
CDs/BiOI	Uniform layered structure nanoplates	Hydrothermal	150-W Xe (vis) (λ = 420 nm)	MO: 98%/50 min	[109] (2016)
CDs/Bi_2_O_2_CO_3_	Nanoparticles /flower-like nanosheets	Dynamic- adsorption precipitation	400-W metal halide (vis) (λ > 400 nm)	MB: 94.45%/120 min Phenol: 61.46%/120 min	[110] (2018)

^1^ MO: methyl orange; BPA: bisphenol A; TC: tetracycline; RhB: rhodamine B; CIP: ciprofloxacin; TC-HCl: tetracycline hydrochloride; IDM: Indometacin; PNP: p-Nitrotoluene; MB: methylene blue.

**Table 8:** Photodegradation activity of miscellaneous other kinds of CD composites.

Photocatalyst	Structure	Synthesis Method	Light Source	Model Pollutant ^1^	Degradation Efficiency/Time	Ref (Year)
NCDs/g-C_3_N_4_	Composite	Polymerization	350-W Xe (vis) (λ = 420 nm)	IDM	91.5%/90 min	[111] (2017)
CDs/g-C_3_N_4_	Nanocomposite	Electrostatic adsorption	(vis)	MB	> 90%/90 min	[112] (2016)
CDs/g-C_3_N_4_	Heterojunction	Low temperature method	250-W Xe (vis) (λ = 420 nm)	RhB and TC-HCl	RhB: 95.2%/210 min TC-HCl: 78.6%/240 min	[113] (2016)
CDs/g-C_3_N_4_	Heterojunction	Impregnation- thermal	300-W Xe (vis) (λ < 400 nm)	Phenol	100%/within 200 min	[114] (2016)
CDs/carbon nitride	Hybrid composite	High temperature treatment	Infrared light (λ > 800 nm)	MO	90%/4 h	[115] (2015)
CDs/FeOOH	Nanocomposite	Hyrothermal	300-W Xe (vis) (λ > 420 nm)	NO	22%/30 min	[116] (2018)
N-CDs/Ag_3_PO_4_/BiVO_4_	Z-scheme hybrid material	Solvothermal- precipitation	300-W Xe (vis) (λ = 420 nm)	TC-HCl	88.9%/30 min	[117] (2018)
CDs/MoO_3_ /g-C_3_N_4_	Z-scheme microstructure	Calcination	350-W Xe (vis) (λ = 420 nm)	TC	88.4%/90 min	[118] (2018)
CDs/CdSe/rGO	Hybrid nanomaterial	Hydrothermal	350-W Xe (vis)	TC-HCl	90%/60 min	[32] (2017)

^1^ IDM: Indometacin; MB: methylene blue; RhB: rhodamine B; MO: methyl orange; NO: nitrogen oxide; TC-HCl: tetracycline hydrochloride; TC: tetracycline.
